# Prevalence of Overweight and Obesity and Their Impact on Spirometry Parameters in Patients with Asthma: A Multicentre, Retrospective Study

**DOI:** 10.3390/jcm12051843

**Published:** 2023-02-25

**Authors:** Abdullah A. Alqarni, Abdulelah M. Aldhahir, Rayan A. Siraj, Jaber S. Alqahtani, Hams H. Alshehri, Amal M. Alshamrani, Ahlam A. Namnqani, Lama N. Alsaidalani, Mohammed N. Tawhari, Omaima I. Badr, Hassan Alwafi

**Affiliations:** 1 Department of Respiratory Therapy, Faculty of Medical Rehabilitation Sciences, King Abdulaziz University, Jeddah 22230, Saudi Arabia; 2 Respiratory Therapy Department, Faculty of Applied Medical Sciences, Jazan University, Jazan 45142, Saudi Arabia; 3 Department of Respiratory Care, College of Applied Medical Sciences, King Faisal University, Al-Ahsa 31982, Saudi Arabia; 4 Department of Respiratory Care, Prince Sultan Military College of Health Sciences, Dammam 34313, Saudi Arabia; 5 Department of Chest Medicine, Faculty of Medicine, Mansoura University, Mansoura 35516, Egypt; 6 Faculty of Medicine, Umm Al-Qura University, Mecca 21514, Saudi Arabia

**Keywords:** obesity, overweight, asthma, lung function, BMI, spirometry

## Abstract

Introduction: Obesity is a common comorbidity in patients with asthma and has a significant impact on health and prognoses. However, the extent to which overweight and obesity impact asthma, particularly lung function, remains unclear. This study aimed to report on the prevalence of overweight and obesity and assess their impacts on spirometry parameters in asthmatic patients. Methods: In this multicentre, retrospective study, we reviewed the demographic data and spirometry results of all adult patients with confirmed diagnoses of asthma who visited the studied hospitals’ pulmonary clinics between January 2016 and October 2022. Results: In total, 684 patients with confirmed diagnoses of asthma were included in the final analysis, of whom 74% were female, with a mean ± SD age of 47 ± 16 years. The prevalence of overweight and obesity among patients with asthma was 31.1% and 46.0%, respectively. There was a significant decline in spirometry results in obese patients with asthma compared with patients with healthy weights. Furthermore, body mass index (BMI) was negatively correlated with forced vital capacity (FVC) (L), forced expiratory volume in one second (FEV_1_), forced expiratory flow at 25–75% (FEF _25–75%_) L/s and peak expiratory flow (PEF) L/s (r = −0.22, *p* < 0.001; r = −0.17, *p* < 0.001; r = −0.15, *p* < 0.001; r = −0.12, *p* < 0.01, respectively). Following adjustments for confounders, a higher BMI was independently associated with lower FVC (B −0.02 [95% CI −0.028, −0.01, *p* < 0.001] and lower FEV_1_ (B −0.01 [95% CI −0.01, −0.001, *p* < 0.05]. Conclusions: Overweight and obesity are highly prevalent in asthma patients, and more importantly, they can reduce lung function, characterised mainly by reduced FEV_1_ and FVC. These observations highlight the importance of implementing a nonpharmacological approach (i.e., weight loss) as part of the treatment plan for patients with asthma to improve lung function.

## 1. Introduction

Asthma, a common pulmonary disease, is characterised by airway hyperresponsiveness, inflammation and remodelling, and it is associated with variable airflow limitation and the presence of respiratory symptoms that vary over time and in intensity. Asthma affects around 300 million people worldwide, and it is expected that closer to 400 million people will have this condition by 2025 [1]. Patients with asthma tend to have variable combinations of pulmonary symptoms, including wheeze, cough, shortness of breath and chest tightness. Worsening of these respiratory symptoms (referred to as exacerbation) may lead to frequent visits to emergency departments and impact overall quality of life [2]. Several factors have been suggested to be associated with the exacerbation of asthma symptoms, one of which is obesity.

Obesity is one of the most common asthma comorbidities [2] and is defined as an excessive accumulation of body fat that leads to a generalised increase in body mass or adipose tissue, which increases the risk of health problems [3]. Body mass index (BMI), calculated as weight in kilograms (kg) divided by the square of height in metres (m^2^), is the most widely used screening tool to determine overweight and obesity [4]. According to the World Health Organisation (WHO), BMI values of between 25 and 29.9 kg/m^2^ are considered to be overweight, while individuals with BMIs of 30 kg/m^2^ and higher are classified as obese [5]. Obesity is further classified by the Centres for Disease Control and Prevention (CDC) into three different categories: class I, or mild (30–34.9 kg/m^2^), class II, or moderate (35–39.9 kg/m^2^), and class III, or morbid (above 40 kg/m^2^) [6].

Obesity is prevalent among adults and children with asthma worldwide [2]. Although the prevalence of obesity in patients with physician-diagnosed asthma is unclear, previous studies have shown that the prevalence of obesity in individuals with self-reported asthma ranges from 15% to 52% [7,8,9]. More importantly, studies suggest that overweight and obesity in conjunction with asthma may lead to deterioration in pulmonary function, which has been shown to be consistent with poor asthma control [10]. In support of this, it has been reported that asthmatic patients who are obese tend to have a four- to six-fold increased risk of hospitalisation compared with non-obese asthmatic patients [11]. Although the exact pathophysiological mechanism remains unknown, it is thought the lung compression caused by accumulation of body fat around the thoracic and abdominal cavities (abdominal obesity) may lead to airway narrowing and increased airway resistance [12]. In addition, it has been suggested that obesity may increase the production of pro-inflammatory mediators that worsen airway inflammation, subsequently causing airway hyperreactivity [13].

Although the impact of obesity on lung function, including spirometry parameters in adults with asthma, has been reported in previous studies, there is still controversy over whether obesity further worsens airway obstruction [7,8,9]. This controversy is likely due to the fact that some previous studies rely on self-reported asthma diagnoses or self-reported height and weight rather than diagnosis by a physician in clinic or measured height and weight [7,8,9]. Thus, further studies are warranted to better understand the impact of obesity on a wide range of spirometry parameters: peak expiratory flow (PEF), forced expiratory volume in one second (FEV_1_), ratio of FEV_1_ to forced vital capacity (FEV_1_/FVC), FVC and forced expiratory flow at 25% and 75% of the pulmonary volume (FEF _25–75%_).

Preliminary reports suggest that both asthma and obesity can lead to worsening of respiratory symptoms and increased risk of hospitalisation. However, the prevalence of obesity and the extent to which overweight and obesity impact lung function, particularly spirometry parameters among patients with asthma in Saudi Arabia, has not been studied. Therefore, this study aimed to report on the prevalence of overweight and obesity and assess their impacts on spirometry parameters in asthmatic patients.

## 2. Materials and Methods

### 2.1. Study Design and Settings

This multicentre, retrospective study was conducted to investigate the impact of overweight and obesity on spirometry parameters among patients with asthma. The data collection process was carried out between 1 April 2022 and 31 October 2022 at King Abdulaziz University hospital and two Ministry of Health hospitals in Saudi Arabia.

### 2.2. Study Population

We retrospectively reviewed the electronic medical records of 1156 outpatients with confirmed asthma diagnoses who had scheduled visits and consultations with specialists and were treated between 1 January 2016 and 31 October 2022. We collected spirometry results and demographic data (e.g., height, weight, BMI, age, gender and smoking status). Demographic data were collected at the time spirometry was performed. Only patients with multidisciplinary-team-confirmed diagnoses of asthma made in accordance with current nationally and internationally accepted criteria were included in the current study [14]. In the final analysis, we only included asthmatic patients with acceptable and reproducible lung function tests at or after age 18, as well as patients without smoking history due to the difficulty in separating asthma from chronic obstructive pulmonary disease in smokers.

### 2.3. Spirometry Parameters

Only spirometry tests performed in accordance with the current American Thoracic Society/European Respiratory Society guidelines were included in the current study [15]. All spirometry tests were performed in pulmonary clinics by trained pulmonary function technologists. Although the pulmonary function tests were routinely validated by a respiratory consultant, all spirometry tests used in the present study were manually reviewed by two trained senior respiratory therapists (A.A.A. and A.M.A.). The results were not included in the final analysis if the spirometry tests were not acceptable and reproducible. The included spirometry results were obtained using a Sensor Medics Vmax 22 machine (SensorMedics Inc., Anaheim, CA, USA). The following spirometry parameters were recorded and included in the current study: FVC, FEV_1_, ratio of FEV_1_/FVC, FEF _25–75%_ and PEF. If the patient had more than one spirometry test performed between 1 January 2016 and 31 October 2022, only the most recent result was included.

### 2.4. Body Mass Index

Height and weight were routinely measured in the clinics, with patients barefoot and wearing light clothing, using a medical scale (Adam Equipment Inc., Oxford, CT, USA). We only collected BMI values based on the heights and weights measured before spirometry was performed. All asthmatic patients included in the current study were divided into five groups in accordance with WHO and CDC classifications: (1) patients with BMI values of 18.5 to 24.9 kg/m^2^ (lean or healthy weight); (2) patients with BMI values of 25 to 29.9 kg/m^2^ (overweight); (3) patients with BMI values of 30 to 34.9 kg/m^2^ (mild or class I obesity); (4) patients with BMI values of 35 to 39.9 kg/m^2^ (moderate or class II obesity); and (5) patients with BMI values of 40 kg/m^2^ or above (morbid or class III obesity) [5,6].

### 2.5. Ethical Considerations

Prior to the start of this study, ethical approval (HA-02-J-008) was obtained from the Unit of Biomedical Ethics Research Committee at the Faculty of Medicine in King Abdulaziz University, Saudi Arabia.

### 2.6. Statistical Analysis

In this study, Stata (version 16) was used for data management and analysis. Figures were generated using GraphPad Prism (version 9). The results are presented as numbers (%) and arithmetic means ± SD for categorical and continuous variables, respectively, unless otherwise stated. The normality of the data was graphically assessed. A one-way ANOVA was performed to compare the mean differences between lean, overweight and specifically classed obesity groups. This was followed by an unpaired Student’s *t* test to compare the mean differences between the two independent data sets. The correlation of BMI with spirometry measures (FEV_1_, FVC, FEF _25–75%_ and PEF) was determined using Pearson’s correlation coefficient. A multiple linear regression model was also performed to determine the factors associated with spirometry measures. *p* < 0.05 was regarded as statistically significant.

## 3. Results

### 3.1. Patient Characteristics

**Figure 1:**
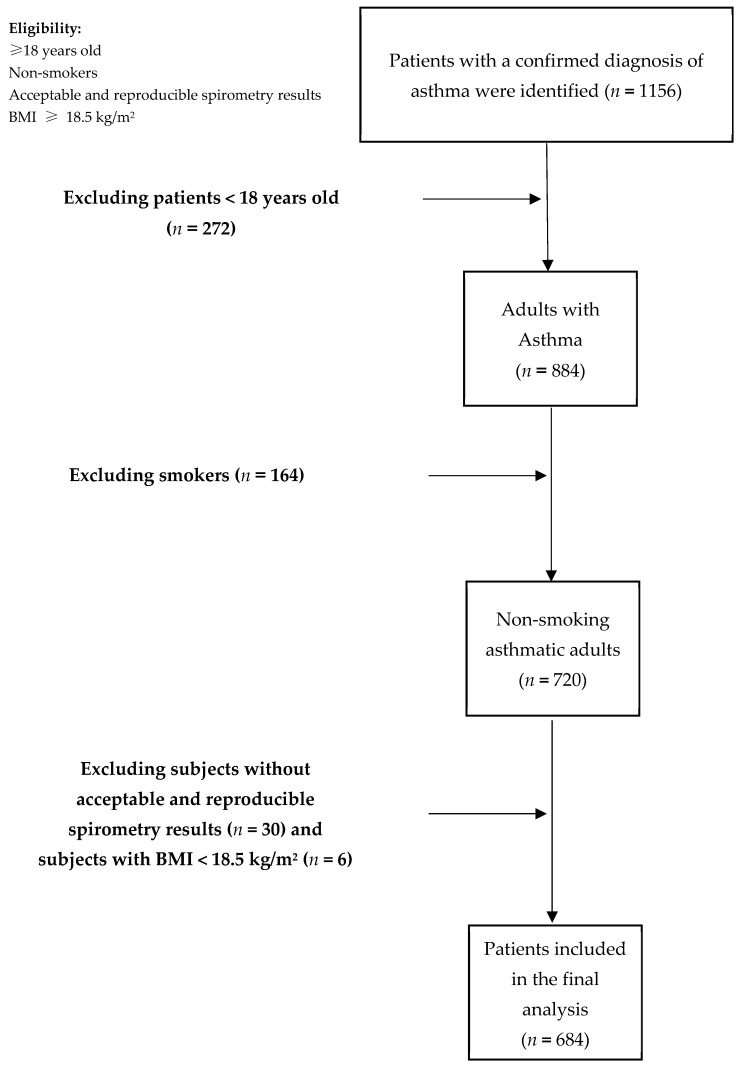
Flow chart of the study.

In total, 1156 subjects with confirmed asthma diagnoses were identified from the databases. After excluding patients <18 years old, smokers and those with BMIs <18.5 kg/m^2^ or without acceptable spirometry results, a total of 684 asthma patients met our inclusion criteria and were included in the final analysis (Figure 1).

The mean ± SD age of the study population was 47 ± 16 years, and there were more females (74%) than males. Of the 684 included patients, 23% had BMIs between ≥18.5 and <25 kg/m^2^ (lean or healthy weight), 31% had a BMIs between ≥25 and <30 kg/m^2^ (overweight) and 46% had BMIs of ≥30 kg/m^2^ (all three classes of obesity). The prevalence of obesity alone and obesity including overweight in patients with asthma was 46% and 77%, respectively. The mean ± SD BMI was statistically significantly greater in females than males (30.5 ± 6.9 vs. 28.9 ± 6.1; *p* < 0.05). The proportion of female patients was higher in the overweight (75%) and obesity (87%) groups compared with lean subjects (66%). The baseline characteristics for the study population are shown in Table 1.

### 3.2. Associations between BMI and Spirometry Parameters

**Figure 2:**
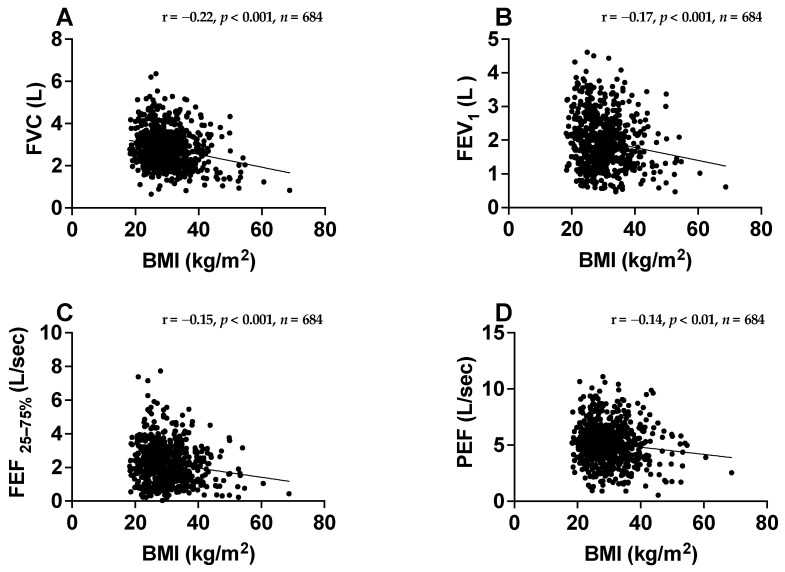
Correlation between body mass index (BMI) and spirometry values among patients with asthma. Correlations between BMI kg/m^2^ with forced vital capacity (FVC) L (**A**), forced expiratory volume in one second (FEV_1_) L (**B**), forced expiratory flow at 25% and 75% of the pulmonary volume (FEF _25–75%_) L/s (**C**) and peak expiratory flow (PEF) L/s (**D**) were assessed.

Pearson correlation analyses were performed to determine whether BMI was associated with spirometry parameters. The results showed that there were statistically significant inverse correlations between BMI and FVC (r = −0.22, *p* < 0.001), FEV_1_ (r = −0.17, *p* < 0.001), FEF _25–75%_ (r = −0.15, *p* < 0.001) and PEF (r = −0.14, *p* < 0.01) (Figure 2).

### 3.3. Impact of Overweight and Obesity on Spirometry Parameters

**Figure 3:**
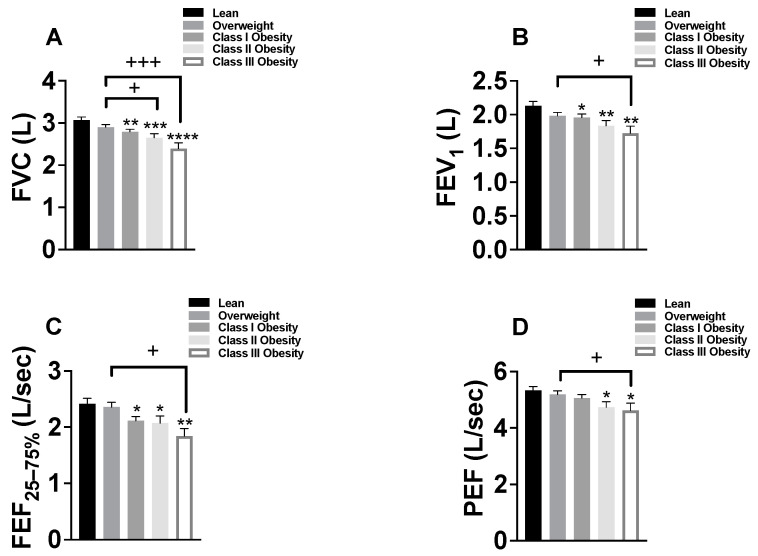
Effect of overweight and obesity on spirometry values in patients with asthma. Patients were divided into four groups based on body mass index (BMI) to assess the impact of overweight and obesity on spirometry values: forced vital capacity (FVC) L (**A**), forced expiratory volume in one second (FEV_1_) L (**B**), forced expiratory flow at 25% and 75% of the pulmonary volume (FEF _25–75%_) L/s (**C**) and peak expiratory flow (PEF) L/s (**D**). Patients with BMIs ≥ 18.5 to 24.9 kg/m^2^ and BMIs of ≥25 to 29.9 kg/m^2^ were classified as lean (*n* = 157) and overweight (*n* = 213), respectively. Obesity was subsequently classified into three different groups: class I (BMI of ≥30 to 34.9 kg/m^2^, *n* = 179), class II (BMI of ≥35 to 39.9 kg/m^2^, *n* = 82) and class III (BMI of ≥40 kg/m^2^ or greater, *n* = 53). Each data point represents mean ± SEM. * *p* < 0.05, ** *p* < 0.01, *** *p* < 0.001, **** *p* < 0.0001 compared with lean; + *p* < 0.05, +++ *p* < 0.001 compared with overweight.

In order to determine whether a progressive decline in spirometry values was observed as BMI increased, we divided asthmatic patients into four different groups based on their BMI values. A one-way ANOVA showed that the means of the spirometry measures (FVC L, FEV_1_ L, FEF _25–75%_ L/s and PEF L/s) differed significantly according to BMI groups (*p* < 0.0001, *p* < 0.01, *p* < 0.01 and *p* < 0.05, respectively) (Figure 3A–D). Unpaired *t* tests were then performed to assess whether there was a significant difference in each spirometry measure based on the weight category. As shown in Figure 3A, although no difference in the mean FVC was found between normal weight and overweight (3.07 ± 0.06 L and 2.90 ± 0.06 L, respectively), mean FVC was significantly reduced in subjects with class I, class II and class III obesity (2.79 ± 0.06 L, *p* < 0.01, 2.65 ± 0.09 L, *p* < 0.001 and 2.39 ± 0.13 L *p* < 0.0001, respectively) compared with normal weight. In addition, mean FVC was found to be significantly decreased in class II and class III, but not in class I, compared with overweight (*p* < 0.05 and *p* < 0.001, respectively).

Similar to the FVC findings, mean FEV_1_ did not significantly differ between asthmatic patients with normal weight and overweight (2.13 ± 0.06 L and 1.98 ± 0.05 L, respectively) (Figure 3B). However, mean FEV_1_ was found to be significantly lower in all classes of obesity (class I, class II and class III) (1.95 ± 0.05 L, *p* < 0.05, 1.83 ± 0.07 L, *p* < 0.01 and 1.72 ± 0.10 L *p* < 0.01, respectively) compared with normal BMI. When compared with overweight patients, mean FEV_1_ remained unchanged in those with class I and class II obesity but was significantly reduced in asthmatic patients with class III obesity (*p* < 0.05) (Figure 3B). Furthermore, mean FEF _25–75%_ was significantly reduced in class I, class II and class III obesity (2.11 ± 0.07 L/s, *p* < 0.05, 2.07 ± 0.13 L/s, *p* < 0.05 and 1.83 ± 0.13 L/s, *p* < 0.05, respectively) but not in overweight patients as compared with normal weight (2.41 ± 0.10 L/s) (Figure 3C). Interestingly, we found that mean PEF did not differ in patients with overweight and class I obesity but was significantly reduced in asthmatic patients with class I and II obesity (4.73 ± 0.19 L/s, *p* < 0.05 and 4.62 ± 0.26 L/s, *p* < 0.05, respectively) compared with normal weight (5.33 ± 0.14 L/s). It was also interesting to find that PEF only decreased in patients with class III obesity when compared with overweight patients (*p* < 0.05) (Figure 3D).

A multiple linear regression model was performed to assess the independent associations of BMI with spirometry measures. BMI was significantly associated with FVC (L) and FEV_1_ (L) following adjustments for age and gender (adjusted β: −0.02; 95% CI: −0.028 to −0.01; *p* < 0.001) and (adjusted β: −0.01; 95% CI: −0.01 to −0.001; *p* < 0.05), respectively. In addition, simple regression models showed that BMI was associated with FEF _25–75%_ (β: −0.03; 95% CI: −0.04 to −0.014; *p* < 0.001) and PEF (β: −0.03; 95% CI: −0.05 to −0.01; *p* < 0.01). However, the associations were nullified upon further adjustments for age and gender (Table 2).

## 4. Discussion

To the best of our knowledge, this study is the first to determine overweight and obesity prevalence in asthmatic patients and to assess the impact of overweight and obesity on spirometry measures among asthmatic patients in Saudi Arabia. The main findings of the current study showed that the prevalence of overweight and obesity among patients with asthma was 31% and 46%, respectively. Our findings also demonstrated that there was a significant decline in spirometry results (FEV_1_, FVC, PEF and FEF _25–75%_) in obese patients with asthma compared with normal-weight asthma patients. In addition, we found that BMI was negatively correlated with all spirometry measures and that a higher BMI was independently associated with lower FVC and lower FEV_1_. These findings suggest that obesity can reduce lung function, ultimately leading to poor asthma control, and also highlight the importance of using a nonpharmacological approach (e.g., healthy diet and weight loss) as part of the treatment plan for patients with asthma to improve lung function and, ultimately, asthma management and overall quality of life.

Obesity is one of the most common asthma comorbidities and is associated with increased risks of exacerbation and hospitalisation. Our findings that overweight (31%) and obesity (46%) are prevalent in patients with asthma are similar to a previous study reporting that the prevalence of obesity among patients with asthma is 52% in the Netherlands [8] but contrasts with studies demonstrating a low prevalence of obesity (15% and 27%) among asthmatic patients in Taiwan [16] and Norway [9], respectively. This disparity is most likely attributable to the fact that the selection of asthma patients in the previous studies was based on self-reported asthma [9,16], whereas in the current study, only patients with multidisciplinary-team-confirmed diagnoses of asthma carried out in accordance with current nationally and internationally accepted criteria were included. In addition, the fact that the prevalence of obesity has been increasing in recent years among the general population in Saudi Arabia [17] may explain the high obesity prevalence observed in the current study. Despite the differences in prevalence rates, our finding that 77% of asthmatic patients are obese or at risk of obesity (overweight) is alarming and suggests that early identification of obesity and overweight, through a regular screening tool, should be implemented in asthma clinics in order to reduce the risk associated with obesity. Although the mechanism that links asthma with obesity is not fully understood, it has been reported that obesity may be a consequence of asthma maintenance therapies. For instance, evidence suggests that the use of oral and inhaled corticosteroids is associated with increased body weight [18,19]. On the other hand, several studies describe obesity as a risk factor for asthma [20], indicating that obese patients are at a higher risk of developing the condition, leading to a novel disease phenotype (obesity-associated asthma) that requires careful evaluation and management. Further studies are needed to better understand the characteristics of this phenotype and its main underlying mechanisms.

Obesity has been suggested to be associated with an increased asthma exacerbation rate, but it is unclear whether airflow obstruction is directly responsible for the deterioration of asthma symptoms. Our findings demonstrated a reduction in spirometry parameters (FEV_1_, FVC, PEF and FEF _25–75%_) in asthmatic patients with obesity compared with patients with normal weight, suggesting that obesity may ultimately impair lung function in patients with asthma. This is likely due to the fact that obesity can cause mechanical compression of the diaphragm, as well as the chest cavity [13], which may lead to a reduction in lung function. In addition, it has been reported that excess adipose tissue in obese individuals can further increase inflammatory mediators (e.g., interleukin 6) [21], which have been shown to be associated with impaired lung function [22,23].

Although the impact of overweight and obesity on PEF and FEF _25–75%_ values has not been reported, our findings are supported by a previous study that reported a reduction in FEV_1_ and FVC in self-reported asthma subjects with overweight and obesity as compared with normal weight [9]. This is further strengthened by the findings of this study that a higher BMI is independently associated with lower FVC and FEV_1_ even after adjustments for known confounders. A previous study demonstrated that an intensive six-month weight-loss programme was correlated with improvement in FVC and FEV_1_ in women with BMIs >30 kg/m^2^ [24]. Our findings, together with these observations, suggest that obesity can cause a decline in lung function, which can be reversed by weight loss. Thus, a screening tool to identify asthmatic patients at high risk for obesity should be implemented in order to improve overall quality of life in patients with asthma.

It is also worth noting that we demonstrated that obesity and overweight, in general, are more prevalent among female asthmatic patients (75% and 77%, respectively) than male patients. This finding is supported by a previous study conducted in the US, showing that overweight and obesity are more prevalent in females with asthma (63% and 82%, respectively) than in males [23]. Overweight and obesity have also been reported to be more prevalent in females (54% and 66%, respectively) than males in Norwegian patients with self-reported asthma [9]. Our findings, together with these previous observations, may be explained by the fact that the proportion of asthma, regardless of subjects’ body weight and geographical location, is found to be higher in female than male subjects in the current study (74% prevalence), as well as in a previous study (65% prevalence) [25]. In addition, obesity without asthma has been reported to be higher in female than male subjects. A high rate of asthma with obesity in adult women suggests that sex hormones and nutrition quality may play a role in the presence and severity of asthma in obese patients.

### 4.1. Strengths

This study has a number of strengths. First, most studies have assessed the impact of overweight and obesity on lung function in patients with self-reported asthma. The current study only included patients whose diagnoses of asthma were made and confirmed in accordance with current nationally and internationally accepted criteria. Second, we only included patients with spirometry tests performed in accordance with the current American Thoracic Society/European Respiratory Society guidelines. In addition, two trained respiratory therapists reviewed all spirometry tests and further excluded tests that were not acceptable and reproducible. Third, some previous studies have relied on self-reported height and weight. In the current study, BMI was calculated based on height and weight measured using medical scales in pulmonary clinics under the supervision of a trained nurse or respiratory therapist.

### 4.2. Limitations

The current study is not without limitations. First, we were unable to study lung volumes and diffusion capacity as these tests are either unavailable or not routinely performed in our pulmonary clinics for patients with asthma. Second, it is known that the use of asthma maintenance therapies (e.g., inhaled corticosteroids) can lead to better asthma control and improvement in asthma symptoms and lung function. In the current study, all patients were on inhaled corticosteroids as a maintenance therapy to control asthma symptoms. However, it remains critical in the current study to determine the doses of inhaled corticosteroids, the levels of patient adherence to therapies and whether those patients were on other asthma control therapies due to the unavailability of these data. Thus, it is important to acknowledge that spirometry parameters can also be affected by patient non-adherence to therapies and/or types of therapy added on to existing inhaled corticosteroid treatment. Third, the prevalence of overweight and obesity was assessed in this study based on BMIs calculated from height and weight measured before spirometry was performed. Although BMI is currently considered to be the gold standard and is used by international organisations (e.g., WHO and CDC) to classify overweight and obesity, it should be noted that it lacks the ability to differentiate between fat and lean mass and does not take into account the differences in fat distribution. This is unlikely to affect the results of this study, as previous studies have shown that abdominal and thoracic fat have a differential effect on lung volumes [26]. There is no evidence to suggest that spirometry parameters are affected by differences in fat distribution. Fourth, our entire study population was diagnosed with asthma in Saudi Arabia. Thus, the findings may not translate to individuals with other chronic pulmonary and non-pulmonary diseases and/or other ethnic groups.

### 4.3. Practical Implementation

Obesity is an asthma comorbidity that can eventually contribute to worsening respiratory symptoms. We report here that the prevalence of obesity and overweight in asthmatic patients is high, and that obesity can lead to a reduction in lung function in patients with asthma. In addition to pharmacological therapies, our findings highlight the importance of using non-pharmacological add-on therapies (e.g., physical exercise, healthy diet and weight loss) as part of the treatment plan for patients with asthma to improve lung function and, ultimately, asthma symptoms and overall quality of life. Further studies are needed to explore the impact of different lifestyle strategies on the treatment of asthma patients.

## 5. Conclusions

Overweight and obesity are highly prevalent in asthma patients, and, more importantly, they can reduce lung function, characterised mainly by reduced FEV_1_ and FVC. These observations suggest that weight loss may reduce the severity of asthma and that early obesity prevention and healthy lifestyles should be implemented through routine screening in primary care to improve lung function, thereby leading to improvements in asthma management, as well as quality of life.

## Figures and Tables

**Table 1:** Patient characteristics (*n* = 684).

Variable	Lean (*n* = 157)	Overweight (*n* = 213)	Class I Obesity (*n* = 179)	Class II Obesity (*n* = 82)	Class III Obesity (*n* = 53)
Age (years)	40 ± 16	47 ± 17	51 ± 15	51 ± 14	53 ± 14
Height (cm)	161 ± 8	163 ± 8	161 ± 10	157 ± 21	156 ± 9
Weight (kg)	59 ± 8	71 ± 8	83 ± 12	90 ± 18	111 ± 16
BMI (kg/m^2^)	22 ± 2	27 ± 1	32 ± 1	37 ± 1	46 ± 6
Female, *n* (%)	104 (66%)	160 (75%)	130 (73%)	69 (84%)	44 (83%)

Data are represented as mean ± SD unless otherwise stated. BMI: body mass index. Obesity is classified based on BMI according to the World Health Organization classification: class I = 30–34.9 kg/m^2^, class II = 35–39.9 kg/m^2^ and class III = 40 kg/m^2^ and greater.

**Table 2:** Analysis of the associations of BMI with spirometry measures.

Independent Variable		BMI
	β (95% CI; *p*-Value)	Adjusted β (95% CI; *p*-Value)
FVC L	−0.03 (−0.04 to −0.02; *p* < 0.001)	−0.02 (−0.028 to −0.01; *p* < 0.001) ^1^
FEV_1_ L	−0.02 (−0.028 to −0.01; *p* < 0.05)	−0.01 (−0.01 to −0.001; *p* < 0.05) ^1^
FEF (25–75%) L/s	−0.03 (−0.04 to −0.014; *p* < 0.001)	−0.012 (−0.025 to 0.001; *p* = 0.058) ^1^
PEF L/s	−0.03 (−0.05 to −0.01; *p* < 0.01)	−0.014 (−0.03 to 0.01; *p* = 0.179) ^1^

^1^ Adjusted for age and gender. FVC: forced vital capacity. FEV_1_: forced expiratory volume in one second. FEF _25–75%_: forced expiratory flow at 25% and 75% of the pulmonary volume. PEF: peak expiratory flow. BMI: body mass index.

## Data Availability

All data generated and analysed during this study are available from the corresponding author upon reasonable request.
